# “A Convenient Memory,” Indeed

**Published:** 1896-06

**Authors:** 


"A CONVENIENT MEMORY" INDEED.—The *Medical News* for May, [McL.] says of the much discussed bill now agitating the profession, referred to *copiously* elsewhere, that it is the only bill that has been acted on and approved by the State Medical Association; that the profession have had no knowledge of other bills that have gone before the legislature, and no opportunity to express an opinion. Now, at Belton, in 1884, when there was a very full meeting, in the palmiest days of the Association, there were two bills presented, one by Cupples, and one by Daniel. Both were read, and the latter mentioned, was, by resolution, ordered printed, 1000 copies, for distribution, and a committee was appointed to go to Austin and personally present and urge it. The committee was composed of Cupples, Tyner, Chew, McLaughlin and Daniel. What's the matter with the doctor's memory? "Convenient?" Not at all.

